# Ear Rachis Xylem Occlusion and Associated Loss in Hydraulic Conductance Coincide with the End of Grain Filling for Wheat

**DOI:** 10.3389/fpls.2016.00920

**Published:** 2016-06-27

**Authors:** Hayet Neghliz, Hervé Cochard, Nicole Brunel, Pierre Martre

**Affiliations:** ^1^UMR GDEC, INRA, Blaise Pascal UniversityClermont-Ferrand, France; ^2^Laboratoire d’Ecophysiologie Végétale, Ecole Normale SupérieureKouba, Algeria; ^3^UMR PIAF, INRA, UCAClermont-Ferrand, France

**Keywords:** grain development, grain maturation, hydraulic conductance, wheat (*Triticum aestivum* L.), xylem embolism, xylem occlusion

## Abstract

Seed dehydration is the normal terminal event in the development of orthodox seeds and is physiologically related to the cessation of grain dry mass accumulation and crop grain yield. For a better understanding of grain dehydration, we evaluated the hypothesis that hydraulic conductance of the ear decreases during the latter stages of development and that this decrease results from disruption or occlusion of xylem conduits. Whole ear, rachis, and stem nodes hydraulic conductance and percentage loss of xylem conductivity were measured from flowering to harvest-ripeness on bread wheat (*Triticum aestivum* L.) cv. Récital grown under controlled environments. Flag leaf transpiration, stomatal conductance, chlorophyll content and grain and ear water potentials were also measured during grain development. We show that grain dehydration was not related with whole plant physiology and leaf senescence, but closely correlated with the hydraulic properties of the xylem conduits irrigating the grains. Indeed, there was a substantial decrease in rachis hydraulic conductance at the onset of the grain dehydration phase. This hydraulic impairment was not caused by the presence of air embolism in xylem conduits of the stem internodes or rachis but by the occlusion of the xylem lumens by polysaccharides (pectins and callose). Our results demonstrate that xylem hydraulics plays a key role during grain maturation.

## Introduction

In cereals, once the final grain number and grain size potential has been established, a few days after anthesis (Yang et al., 2009), grain yield is largely determined by the duration of the grain-filling phase (Triboi and Triboi-Blondel, 2002; Borrás and Gambín, 2010). The major effect of water deficit or high temperature during the post-anthesis period on the accumulation of grain dry mass and final grain yield is to reduce the duration of the grain-filling period (Panozzo and Eagles, 1999; Dupont et al., 2006). It is thus important to better understand the physiological mechanisms leading to the arrest of grain filling in cereals. Early studies of the uptake of ^14^C assimilates into developing wheat and barley (*Hordeum vulgare* L.) grains indicated that it is unlikely that lack of assimilate is responsible for the termination of starch (which accounts for 70–80% of final grain dry mass) synthesis in the endosperm (Jenner and Rathjen, 1975, 1977; Cochrane, 1985), and wheat canopy can maintain high leaf nitrogen concentration and photosynthetic capacity until end of grain filling when nitrogen is well managed (Triboi and Triboi-Blondel, 2002). Therefore, unless plants are under severe nitrogen deficit, whole plant senescence in monosporic plants such as wheat does not drives grain filling dynamics.

The development of orthodox seeds (i.e., desiccation tolerant seeds), including seeds of most grass species, from fertilization to ripeness maturity can be divided into three main phases: the lag phase, the effective grain-filling phase and the maturation-drying phase (Bewley and Black, 1985; Sabelli and Larkins, 2009). The lag phase is a period of active cell division and differentiation. It is characterized by a rapid increase of the mass of water per grain (hereafter grain water content) with negligible gains in dry matter (Saini and Westgate, 2000). Following the lag phase is a period of rapid dry matter accumulation resulting from the deposition of carbon (starch or oil) and nitrogen (protein) reserves. Grain water content reaches its maximum value at the beginning of this phase and stays nearly constant until the end, while grain water concentration decreases steadily during the first two phases of grain development and is closely related to the stage of grain development. This apparent desiccation does not affect grain water relations, since osmotic volume (i.e., the cellular volume on which osmolytes are diluted) remains fairly constant (Barlow et al., 1980; Bradford, 1994). In the third phase, grains achieve their maximum dry mass, commonly referred to as physiological maturity, and their water content and water and osmotic potentials decrease gradually, which results in a reduction of metabolism and the embryo enters a metabolic inactive or quiescent state (Saini and Westgate, 2000).

Some authors have suggest that grain dehydration induces a rapid decrease of grain osmotic potential that may inhibit grain metabolism and cause the end of grain filling (Barlow et al., 1980; Westgate and Boyer, 1986; Saab and Obendorf, 1989; Egli, 1990; Westgate, 1994). Other results suggested that the end of grain filling could rather be caused by steric hindrance in the endosperm due to the accumulation of starch granules. This hypothesis is supported by the many reports showing that the end of grain filling occurs at a critical grain water concentration (Ellis and Pieta Filho, 1992; Calderini et al., 2000; Borras et al., 2004; Borrás and Gambín, 2010; Ferreira et al., 2012). For wheat this water concentration is close to 46%. As noted by Ferreira et al. (2012), it corresponds to the water concentration of closely packed wheat starch granule (related to the fractional solid content) determined by Willett (2001). These authors concluded that the synthesis of starch continue until random close packing of starch granule is reached at around 46% grain water concentration.

The attainment of maximum grain dry mass coincides with the onset of a rapid decline in grain water content (Sofield et al., 1977a; Barlow et al., 1980; Nicolas et al., 1985; Schnyder and Baum, 1992). Although wheat, maize (*Zea mays* L.) and soybean (*Glycine max* L.) reach physiological maturity at different grain water concentration (ranging from 33% for maize to 59% for soybean), for all three species this critical water concentration corresponds to a water potential of the embryo/axis close to -1.6 MPa (Egli and TeKrony, 1997). These results suggest that changes in grain water relations may be a cause rather than a consequence of the cessation of grain dry mass accumulation. Accordingly, several authors proposed that the cessation of grain growth is triggered by a rapid decrease of grain osmotic potential (Saab and Obendorf, 1989; Egli, 1990; Westgate, 1994).

Few reports on the mechanism and route of water loss from cereal grains have been published. According to some authors, water is lost primarily by evaporation from the surface of surrounding grain structures due to an increase in the permeability of the pericarp (Radley, 1976; Lee and Atkey, 1984). Water may also move from the grain to the parent plant thanks to a metabolically active process (Meredith and Jenkins, 1975). However, the relocation of water from the seed to the parent plant (back flow) is not the means by which the water loss occurs in orthodox seeds (Kermode and Finch-Savage, 2002). Knowledge of changes in hydraulic conductance is required to determine whether xylem dysfunction is responsible for declining xylem flows into the grain at physiological maturity.

Research efforts have focused so far mainly on the physiological consequences of grain dehydration on grain growth and several hypotheses may explain how it may impair grain filling. The mechanisms triggering the sudden grain dehydration at the end of the filling phase have received much less attention. The objective of our study was to unravel these mechanisms in wheat. Our analysis is based on the timing of the successive physiological modifications occurring after anthesis. As far as, we know there are no reports in the literature of the changes in hydraulic properties of cereal inflorescence during their development. Therefore, the present work is an attempt to find out how hydraulic conductance in wheat stem internodes, intact ear, rachis and grains change during development, and to investigate the relationship between hydraulic conductance and water loss during grain maturation. We hypothesize that (1) water flow through the xylem declines markedly during the latter stages of grain development; and (2) that this reduction results from embolism or xylem occlusion in the rachis and/or in the stem.

## Materials and Methods

### Plant Material and Growth Conditions

Seeds of the bread wheat (*Triticum aestivum* L.) cv. Récital were sown in 50 mL plastic pots (two seeds per pot) filled with a peat moss mix and were kept in a greenhouse for 2 weeks. Light/dark air temperatures in the greenhouse were controlled at 18°C/10°C and air relative humidity at 70%/50% (light/dark). The plants were then transferred into a vernalization growth chamber maintained at 4°C ± 1°C, the PPFD (photosynthetic photon flux density) at the top of the plants was 43 mmol m^-2^ d^-1^ during the 8 h photoperiod, and the relative humidity was maintained at 40%. After 8 weeks, the plants were transplanted into polyvinyl chloride (PVC) columns (1.9 L, i.d. 7.5 cm, length 50 cm) filled with a 1:3 (v:v) mixture of river sand:peat moss mix (two plants per column) and were arranged in a 9 m^-2^ walk-in growth chamber to form a homogeneous stand with a plant density of 261 plant m^-2^. The air temperature in the growth chamber was 20°C/16°C (light/dark), the PPFD, provided by metal halide lamp (HQI-TS 400W/NDL, Osram, München, Germany), averaged 550 mmol m^-2^ s^-1^ during the 16 h photoperiod and the air relative humidity was controlled at 70%/50% (light/dark). The plants were watered with 34 mL pot^-1^ d^-1^ until growth stage 31 (Zadoks et al., 1974), and 68 mL pot^-1^ d^-1^ afterward, of tap water using an automated drip feed system. Once a week, the water was replaced by a 0.2 M NH_4_NO_3_ solution. Starting at heading, air temperature next to the ears was measured using insulated Cu/Co thermocouples arranged to form a 0.5 × 0.5 m grid and recorded every 5 min on a CR1000 datalogger (Campbell Scientific Ltd., Leicester, UK). Three independent experiments were carried out in different period of the year. In each experiment, each trait was measured on three to five plants on each sampling date and was averaged for statistical analyses.

Ears were tagged with the date on which 50% anthers were exerted and timing of all subsequent operations was related to this date. All measurements except xylem embolism were carried out every 8 days from anthesis to ripeness maturity. All measurements and samples were taken 4 h after the beginning of the light period to avoid any diurnal effect. At each sampling date, three plants of the same developmental age were harvested for measurements. One plant was used for measuring flag leaf and ear water potential and water content, and flag leaf chlorophyll concentration and transpiration. The second one was used for measuring ear and rachis hydraulic conductance. The third plant was used to determine xylem embolism.

### Flag Leaf Chlorophyll Content, Transpiration, Water Potential and Water Content

Flag leaf chlorophyll concentration was non-destructively measured using a hand-held SPAD-502 chlorophyll meter (Minolta Camera Co., Osaka, Japan). Average SPAD chlorophyll readings were calculated from three measurements taken equal distances from the tip to the base of the leaf lamina. Transpiration rate (*E*, mmol m^-2^ s^-1^) of the flag leaf lamina was measured using a LI-1600 steady-state porometer (LI-COR Inc., Lincoln, NE, USA). Measurements were taken on both sides of the lamina and the sum of the measurements was used.

Mid-day water potential of the flag leaf (Ψ_leaf_, MPa) was determined using a Scholander-type pressure chamber following Boyer (1995) recommendations. After the balance pressure was determined, the leaf fresh mass was immediately determined and dry mass was determined after oven-drying at 80°C to constant mass. The difference between fresh and dry mass was taken as the water content of the sample and water concentration was calculated as 100 × water content divided by fresh mass.

### Ear and Grain Water Potential and Water Content

The entire ear was removed from the plant, and its water potential was measured using the Scholander-type pressure chamber as described for flag leaves. After the balance pressure was determined, the ear was placed in an air-tight plastic bag and immediately transferred in a humid box where the two basal grains of the middle spikelets from the two sides of the ear were excised and sealed in C-52 psychrometer chambers (Wescor Inc., Logan, UT, USA). The psychrometer chambers were then placed in an insulated box maintained at 20°C and water potential was measured in dew-point mode every 20 min until equilibrium was reached (usually in 1 h for bracts and 4 h for grains) using a programmable PsyPro water potential system (Wescor). The remaining grains were separated and their fresh and dry mass was determined and their water content and concentration calculated as described for flag leaves.

### Ear, Rachis and Grain Hydraulic Conductance

**FIGURE 1:**
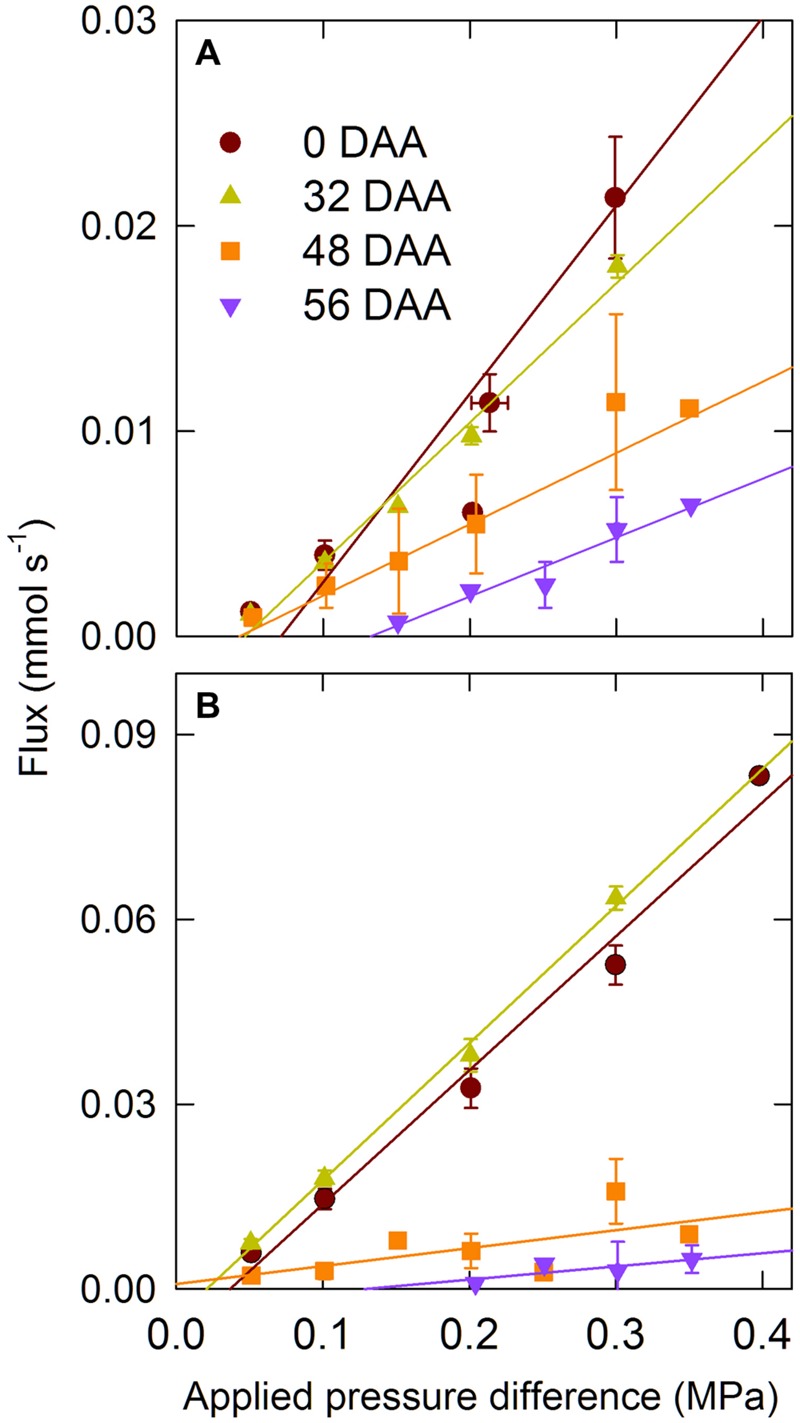
**Water flux versus applied pressure difference for whole ear **(A)** and rachis **(B)** for the winter bread wheat (*Triticum aestivum* L.) cv.** Récital grown under controlled conditions. DAA, days after anthesis. Data are means ± 1 s.e. for *n* = 3 to 5 independent replicates.

Hydraulic conductance of the intact ear (*L*_P,E_, MPa s mol^-1^) and rachis (*L*_P,R_, MPa s mol^-1^) were measured using a Xyl’em apparatus (Bronkhorst, Montigny-Les-Cormeilles, France) equipped with a 20 g h^-1^ full scale flow meter (Cochard et al., 2005, 2007). The ear peduncle was cut with a razor blade at 10 cm from the ear base and immediately put in a bucket of water and returned to the laboratory where it was trimmed under water 1 cm from the base of the ear with a fresh razor blade. The remaining piece of the ear peduncle was kept under water, wrapped with Teflon tape, and then inserted into Exacanal silicon tubing and connected to the Xyl’em tank containing a filtered (0.2 μm) and degassed 20 mM KCl and 2 mM CaCl_2_ solution. The hydrostatic pressure in the tank was increased to 0.05 MPa; after the flow rate was stabilized, usually within 20 min, the hydrostatic pressure was then increased by 0.05 MPa steps to 0.3 MPa, and the flow rate was recorded at each pressure after it stabilized in less than 10 min. After *L*_P,E_ was measured, spikelets were removed under water and *L*_P,R_ was measured as described for the whole ear. Also the flux versus applied pressure difference was linear for all the sampling dates, the pressure needed to get a measurable flux increased with the age of the ear (**Figure 1**). Therefore, *L*_P,E_ and *L*_P,R_ were calculated as the average of the ratio between the water flux and the applied pressure difference at 0.05 and 0.1 MPa.

Grains represent the last component of a serial hydraulic pathway. Thus, the reciprocal of *L*_P,E_ equals the sum of the reciprocal of *L*_P,R_ and the hydraulic conductance of the grains (*L*_P,G_), so the *L*_P,G_ was calculated as:

(1) $$L_{P,G}=\frac{1}{\frac{1}{L_{P,E}}-\frac{1}{L_{P,R}}}$$

The whole spikelets were removed to measure *L*_P,R_, thus *L*_P,G_ also includes the hydraulic conductance of the bracts (palea and lemma), of the glumes, and of most of the rachilla. Therefore, strictly speaking *L*_P,G_ corresponds to the spikelet hydraulic conductance.

### Initial and Maximum Xylem Hydraulic Conductivity of Ear Rachis and Stem Internodes

The main stem was cut at its base and the cut end was placed in water for transport to the laboratory. The ear was trimmed 1 cm from the base of the rachis under water and the spikelets were removed. The stem was cut under water and 1-cm-long segments containing one node were taken. The base of the rachis and the stem segments were wrapped with Teflon tap and fitted into Exacanal silicon tubing connected to three-way stopcocks connected to the Xyl’em apparatus. The initial conductivity (*K*_i_, mmol m s^-1^ MPa^-1^) of each sample was measured with an applied pressure difference of 5 kPa, then samples were perfused once at a pressure of 0.1 MPa for 10 s to dissolve and expel air bubbles. Hydraulic conductivity of each sample was measured again, and flushes were repeated (for 15 s then 1 min) until a maximum conductivity, (*K*_max_, mmol m s^-1^ MPa^-1^) was obtained. Axial hydraulic conductivities (*K*_i_ and *K*_max_) were calculated by dividing the value of the conductances (mml s^-1^ MPa^-1^) by the length (m) of the samples used for the measurements.

### Rachis Anatomy

Ear rachis sampled at 0, 25, 40, 45, and 55 DAA were cut transversally into 10-mm long segments and fixed at 4°C for 5 h in a solution containing 3.7% (v:v) formaldehyde, 50% (v:v) ethanol, and 5% (v:v) acetic acid. Samples were then dehydrated through graded series of ethanol (50%, 70%, 80%, 95%, 100%, and 100% for 30 min each and 100% overnight at 4°C) and finally embedded in L.R. White resin (Sigma–Aldrich, St. Louis, MO, USA). Two to 3 μm thick transverse sections were then cut using an OmU2 rotary microtome (Reichert, Vienna, Austria) equipped with glass knives.

To investigate anatomical features, transverse sections were stained with 0.5% (w:w) toluidine blue O in 2.5% sodium carbonate buffer (pH 11; O’Brien and McCully, 1981). Sections to be examined for polysaccharides were stained with periodic acid-Shiff’s reagent (Ruzin, 1999). To avoid false positive results from the aldehyde fixation, some sections were treated with Schiff’s reagents without a previous treatment with periodic acid as a control of the periodic acid-Schiff’s treatment. Pectins were detected by staining section overnight with freshly prepared 1% (w:w) ruthenium red in distilled water (Vallet et al., 1996). Some sections were also stained overnight in 1% (w:w) lacmoid blue in 3% (v:v) acetic acid to detect callose (Jensen, 1962). Stained sections were dried, mounted in Eukitt (Sigma–Aldrich, St. Louis, MO, USA) and observed with an Axioplan 2 microscope (Zeiss, Jena, Germany). Photomicrographs were taken with an AxioCam HR digital camera (Zeiss) and analysed with the Axiovision digital imaging software (Zeiss).

### Estimation of Grain Filling Duration and of the Onset of the Decrease of Whole Ear, Grain, and Rachis Hydraulic Conductance

Grain filling duration was estimated using a 3-parameter logistic function equation (Triboi et al., 2003).

(2) $$Q(t)=\frac{Q_{\max}}{1+0.05\exp\left(\frac{-4r\left(t-t_{95}\right)}{Q_{\max}}\right)}$$

where *Q* is the quantity of dry mass per grain, *t* is the number of DAA, *Q*_max_ is the final value of *Q* approached as t →-∞, *r* is the maximum rate of accumulation defined as the derivative of the point of inflecion, and *t*_95_ is the duration of accumulation of grain dry mass defined as the duration, from anthesis, in which 95% of *Q*_max_ is accumulated.

The onset of the decrease of whole ear, grain and rachis hydraulic conductance was estimated by fitting a reparametrized form of Eq. [1]:

(3) $$L(t)=L_{\min}+\frac{L_{\max}-L_{\min}}{1+\left(\frac{L_{\max}-L_{\min}}{0.95L_{\max}-L_{\min}}-1\right)\exp\left(\frac{-4r\left(t-t_{05}\right)}{L_{\max}-L_{\min}}\right)}$$

where *L* is the whole ear, rachis, or grain hydraulic conductance, *L*_max_ is the maximum value of *L* approached as t →-∞, *t*_05_ is the DAA at which *L* has decreased by 5% of it is maximum value. The fitted duration of grain filling and the time at which the whole ear, rachis or grain hydraulic conductance had decreased by 5% were considered as significantly different (α = 0.05) when the confidence interval of *t*_95_-*t*_05_ did not contain zero. Non-linear regression and the 95% confidence intervals of the estimated parameters were calculated using the software package R-3.2.1 for Windows (R Core Team, 2015).

## Results

### Grain Development and Plant Water Relations

**FIGURE 2:**
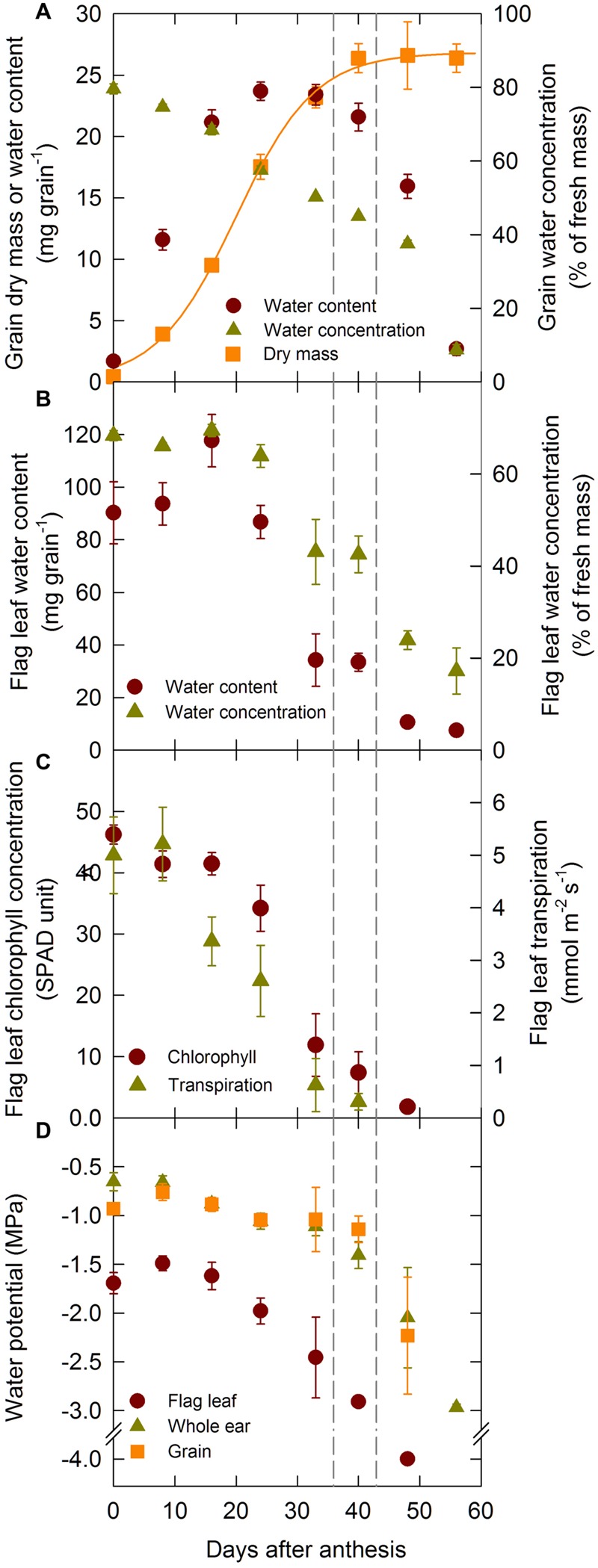
**Grain dry mass accumulation, changes in grain and flag leaf water content, concentration, and changes in flag leaf, whole ear, and grain water potential during grain development for the winter bread wheat cv.** Récital grown under controlled conditions. **(A)** Grain dry mass and water content and concentration versus days after anthesis. **(B)** Flag leaf water content and concentration versus days after anthesis. **(C)** Flag leaf transpiration and chlorophyll concentration versus days after anthesis. **(D)** Whole ear, grain, and rachis water potential versus days after anthesis. The non-linear curves show the results of a three parameter logistic function equation fitted to the grain dry mass data. The vertical dotted lines indicate the 95% confidence interval of the end of grain filling. Data are means ± 1 s.e. for *n* = 3 independent replicates.

The development of orthodox seeds is composed of three distinct phases: lag, grain-filling and drying phases. Under our experimental conditions the lag phase lasted until 16 DAA and the filling phase until 39 DAA (**Figure 2A**). Accordingly, grain dry mass increased steadily during the lag and filling phases and it remained constant thereafter (**Figure 2**). Grain water content increased during the lag phase, remained constant during the filling phase and declined rapidly at the onset of the drying phase. Grain water concentration was maximum at anthesis and decreased continuously during the grain development period.

Flag leaf water content increased slightly during the lag phase of grain development, then decreased rapidly, while flag leaf water concentration was constant until mid-grain filling (24 DAA), then decreased gradually during grain development (**Figure 2B**). Chlorophyll concentration in the flag leaf was maximal and constant during the lag phase (**Figure 2C**), but decreased sharply at the onset of the filling phase as the leaf started to senesce. Leaf senescence was accompanied by a decrease in transpiration (**Figure 2C**). From these results it is clear that leaf senescence starts early during the grain filling period.

**Figure 2D** illustrates the changes in midday leaf (Ψ_L_), ear (Ψ_E_), and grain (Ψ_G_) water potentials during the grain development period. Ψ_G_ and Ψ_E_ were higher (less negative) than Ψ_L_ through the grain development period. Ψ_G_ remained relatively constant and was close to -1 MPa during the lag and filling phases but declined sharply at the onset of the drying phase. After 50 DAA Ψ_G_ was too low to be measured. Ψ_E_ followed a relatively similar pattern to that of Ψ_G_. Ψ_L_ was relatively constant during the lag phase of grain development and began to decrease thereafter as leaves senesced.

### Changes in Hydraulic Conductance with Development

**FIGURE 3:**
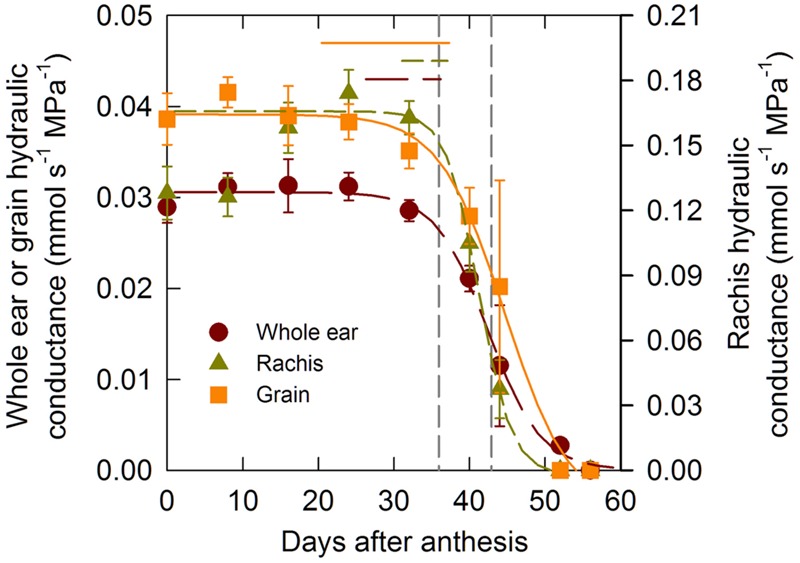
**Changes in whole ear, grain, and rachis hydraulic conductance during grain development for the winter bread wheat cv.** Récital grown under controlled conditions. The non-linear curves show the results of a three parameter logistic function equation fitted to the data. The horizontal lines indicate the 95% confidence interval of the estimated parameter of the logistic function related to the time when the hydraulic conductivity had decreased by 5% of its maximal value. The vertical dotted lines indicate the 95% confidence interval of the end of grain filling. Data are means ± 1 s.e. for *n* = 3 independent replicates.

In order to test the hypothesis according to which water flow through the xylem declines markedly during the latter stages of grain development whole ear hydraulic conductance (*L*_P,E_) and its two additive components (i.e., rachis (*L*_P,R_) and grains (*L*_P,G_) conductances) were measured through grain development. **Figure 3** shows time changes in these three conductances. *L*_P,R_ was four to five-fold higher than *L*_P,E_ and *L*_P,G_ but the trends were comparable across organs. During the lag and the effective filling phases of grain development the conductances were high and roughly constant, although *L*_P,R_ tended to increase during the lag phase. Near the end of the filling phase (between 32 and 40 DAA), the hydraulic conductance of all the organs dropped sharply down to total hydraulic impairment at circa 50 DAA. The comparison of the estimated duration of grain filling and of the time when the whole ear, rachis, and grain hydraulic conductance had decreased by 5% of their initial value confirmed that the whole ear, rachis, and grain hydraulic conductance started decreasing before the estimated end of grain filling (*P* < 0.05).

### Air Embolism

**FIGURE 4:**
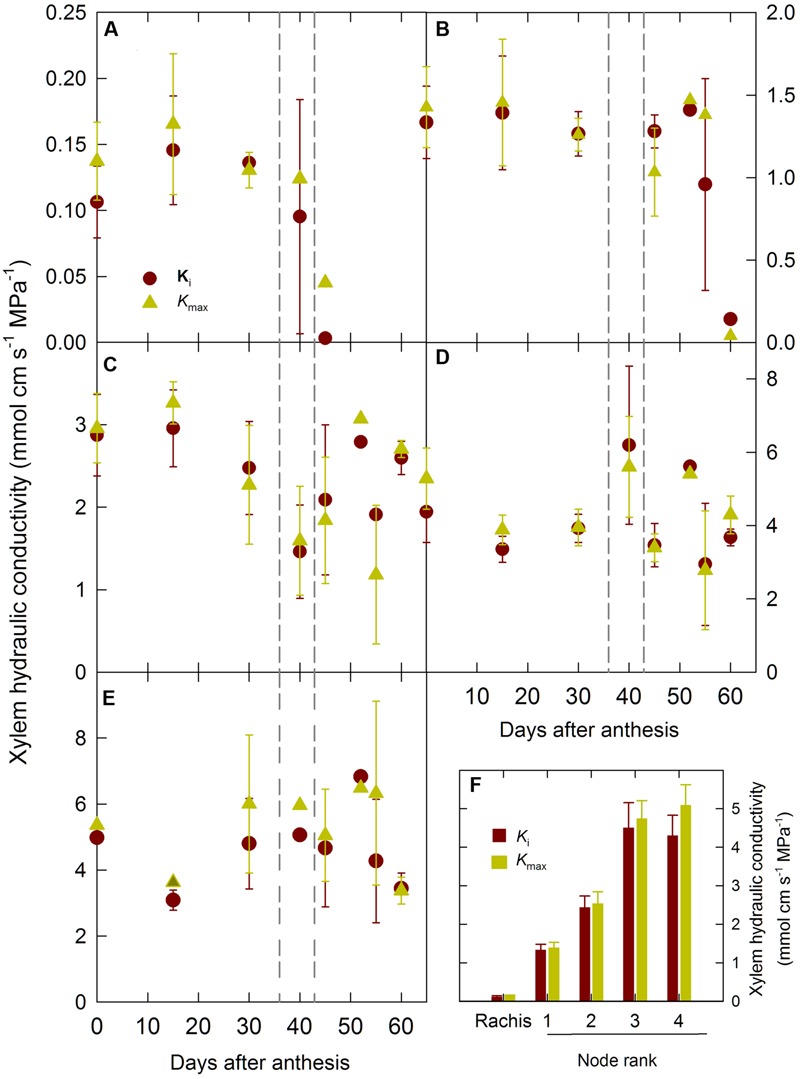
**Changes in initial (*K*_i_) and maximum (*K*_max_) xylem hydraulic conductivity of mainstem internodes and ear rachis during grain development for the winter bread wheat cv.** Récital grown under controlled conditions. **(A)** Ear rachis *K*_i_ and *K*_max_ versus days after anthesis. **(B–E)** Mainstem internodes 1 to 4 (counted basipetally) *K*_i_ and *K*_max_ versus days after anthesis. **(F)** *K*_i_ and *K*_max_ of ear rachis and mainstem internodes averaged between 0 and 40 days after anthesis. The vertical dotted lines indicate the 95% confidence interval of the end of grain filling. Data are means ± 1 s.e. for *n* = 3 independent replicates.

To asses if the drop in hydraulic conductance toward the end of the grain filling was due to air embolism the axial hydraulic conductivity of the rachis and of the stem internodes were measured. **Figure 4** shows the changes in the initial (*K*_i_) and maximum (*K*_max_) axial hydraulic conductivity of the ear rachis and mainstem nodes during the grain development period. *K*_i_ and *K*_max_ of the ear rachis and of the node located at the base of the ear rachis (node 1) were constant until the end of the grain grain-filing period and then dropped to values close to zero after 45–50 DAA (**Figures 4A,B**). *K*_i_ and *K*_max_ of the other mainstem nodes did not show any clear trend during the grain development period (**Figures 4C–E**). In good agreement with hypothesis of plant hydraulic segmentation into regions differing in water transport efficiency (Meinzer et al., 1992; Martre et al., 2001), there was a clear gradient of xylem hydraulic conductivity from the ear rachis to the basal mainstem node (**Figure 4F**). The percent differences between *K*_i_ and *K*_max_ of the ear rachis and all the nodes remained low (≤20%) throughout the grain development period, indicating that there was not significant xylem air embolism during the post-anthesis period (**Figure 4B**).

### Xylem Anatomy

**FIGURE 5:**
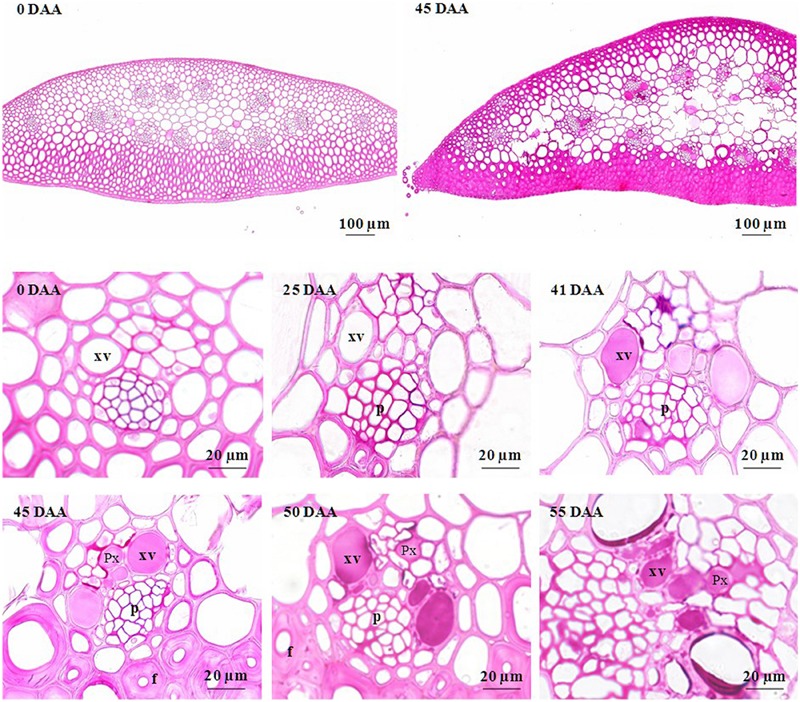
**Photomicrographs of cross sections of developing rachis of the winter bread wheat cv.** Récital grown under controlled conditions. All cross sections were stained with periodic acid-Schiff’s reaction. p, phloem; Px, protoxylem; DAA, days after anthesis; xv, metaxylem.

**FIGURE 6:**
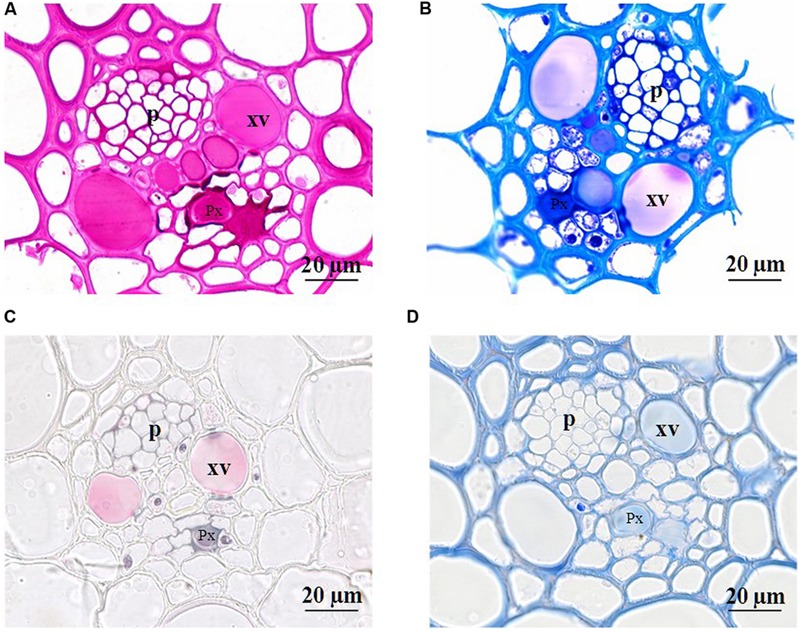
**Photomicrographs of cross sections (2 μm) of developing rachis of the winter bread wheat cv.** Récital sampled at 45 days after anthesis. Cross sections were stained with periodic acid-Schiff’s reaction **(A)**, toluidine blue O **(B)**, ruthidium red **(C)**, and lacmoid blue **(D)**. p, phloem; Px, protoxylem vessel; xv, metaxylem vessel.

To check if the loss of ear hydraulic conductance could results from xylem occlusion in the ear rachis transvers section were examined. **Figure 5** shows transverse sections of ear rachis sampled at various developmental stages and stained with reactions to detect particular compounds. During the lag and filling phases, the xylem lumens were clear of any material. By contrast, during the drying phase these lumens became filled with uniform material. To identify the nature of the substances filling the xylem lumens four histochemical tests were used on rachis sampled during the drying stage (45 DAA; **Figure 6**). Vessel lumens stained purplish red after treatment with the periodic acid-Shiff’s reaction (**Figure 6A**), faint pinkish-purple-blue with toluidine blue O (**Figure 6B**) both indicating the presence of polysaccharides. The use of more specific dyes clearly revealed the presence of pectin and callose (β-1,3-glucan) in the vessel lumens. Pink with ruthenium red (**Figure 6C**), indicating the present of non-methyl-esterified pectins (Vallet et al., 1996) and blue with lacmoid blue (**Figure 6D**) indicating the present of callose (Jensen, 1962).

## Discussion

### Grain Dehydration Is Not Consequence of a General Plant Physiological Disorder Associated with Monocarpic Senescence

Several authors (e.g., Martinez-Carrasco et al., 1988; Schnyder and Baum, 1992; Cochrane et al., 2000; Ferrise et al., 2015) have shown that the end of dry matter accumulation coincides with sudden grain dehydration. Here, we first tested the hypothesis that grain dehydration is a consequence of a general plant physiological disorder associated with its senescence. Our results do not support this hypothesis. Indeed, flag leaf senescence, as monitored by changes in chlorophyll concentration, coincided with the initiation of the grain filling period which is consistent with a reallocation of resources from the source to the sink during grain filling. Similarly, flag leaf water potential and transpiration declined gradually during the grain-filling period and correlated with leaf senescence. Our results give more support to a mechanisms taking place rather at the ear or the grain level. This is illustrated by the decoupling of grain water potential and whole plant water relations as previously reported (Barlow et al., 1980; Fisher and Cash-Clark, 2000).

### Grain Dehydration Is Induced by the Cessation of Water Flow into the Frain

What could cause the sudden grain dehydration at the end of the filling phase? It is the consequence of an unbalance between water lost by evaporation through the grain pericarps, and water entering the grain through the xylem tissue. Near the end of the grain filling phase, the enveloping bracts (lemma and palea) tend to open which may favor grain water loss. However, our measurements (data not shown), suggest that this effect is rather small and cannot explain alone the sharp grain dehydration. Therefore, we have more thoroughly explored in this study the hypothesis of an impairment of the water flow into the grain. We report here for the first time that the onset of grain dehydration is preceded by a sharp decrease of the rachis hydraulic conductance. Our data suggest a basipetal evolution of this hydraulic impairment, with rachis being impacted first and stem nodes last. This observation provides strong support for previous suggestions that grain dehydration is induced by the cessation of water flow into the grain (Sofield et al., 1977b; Cochrane, 1985) and not to the increasing loss of water by evaporation from the surface of the grain (Radley, 1976; Lee and Atkey, 1984).

The independence of water relations between wheat grains and the mother plant (Barlow et al., 1980; Fisher and Cash-Clark, 2000) and the apparent xylem discontinuity in the grain pedicle (Zee and O’Brien, 1970; Cook and Oparka, 1983) has led to suggest that water is imported in the grain through the phloem rather than through the xylem (for a review see Bradford, 1994). However, direct measurements of water flow using nuclear magnetic resonance imaging has shown that longitudinal bulk flow of water within wheat grains are on a too high scale to be due to phloem transport alone (Jenner et al., 1988). In any case, to sustain phloem transport phloem water must flow back to the mother plant through the xylem (Lang, 1990). Therefore, whatever the bulk of water enter in the grains through the phloem (the most likely route) or the xylem, the occlusion of ear rachis xylem as reported in this study would eventually stop phloem transport to the grains. Moreover, the differences of water potential reported here and in previous studies (e.g., Barlow et al., 1980) suggest that water flow between the mother plant and the grains may diurnally reverse, as demonstrated in cowpea (Vigna unguiculata [L.] Walp.; Pate et al., 1985). The occlusion of xylem vessels in the ear rachis would counter reverse water flows during the diurnal period.

### The Deposition of a Pectic Gel in the Lumen of Xylem Vessels in the Ear Rachis Causes a Drop in It Is Hydraulic Conductivity

The reduction in xylem water transport capacity is caused by a physical blockage of the water flow, which may originate from the presence of air bubbles or solid particles in the vessel lumens. Air embolism is well known to lower xylem conductance in plants exposed to extreme water deficit (Sperry and Tyree, 1988). To test this hypothesis, we measured the changes in xylem hydraulic conductance of the ear rachis and stem segments after having purged them with pressurized and degassed water. The impact of this treatment was marginal suggesting that air embolism was not at the origin of this blockage.

To test if xylem occlusion by solid substances was the cause of rachis hydraulic conductance decrease, we observed the lumen content on transverse rachis sections at different ages. A substance progressively plugged the xylem lumens during the drying phase. We used different histochemical stains to identify this substance. The red reaction to periodic acid-Shiff’s indicated the presence of aldehydes, which were probably derived from the oxidation of saccharides by periodic acid. The xylem lumens were thus filled with polysaccharides gels. Furthermore, the substance stained purple with toluidine blue, suggesting its pectic nature. This finding was confirmed by pink coloration observed after treatment with ruthenium red. Similarly, Cochrane (1985) observed that the lumen of xylem vessels in the rachis of wheat and barley ears sampled 8 days after the maximum grain dry mass was achieved (i.e., during the desiccation phase) are filled with pectic material. Here, we show that the deposition of a pectic gel in the lumen of xylem vessels in the ear rachis causes a drop in it is hydraulic conductivity. The earlier decrease in rachis conductance may suggest that this secretion started slightly before the drying phase, but the temporal resolution of our histochemical analysis was not precise enough to confirm this hypothesis. This conclusion is supported by the parallel downward progression of the material present in the lumen of xylem vessels and of the grain water concentration with a maximum of intensity of staining observed at the level of the rachis where the water concentration of the grains was about 50% (Cochrane, 1985).

The positive reaction with lacmoid blue indicated the presence of callose in the material deposited in the lumens of xylem vessels. This observation should be confirmed using other dyes or antibodies but it does not change our main conclusion that xylem vessels are plugged with polysaccharide compounds. Callose has been detected in pits of developing xylem and the callose synthase was detected at the surface of these locations in healthy tissues (Gregory et al., 2002). Callose has also been detected in amorphous plug formed in mature xylem vessels of trees infected by citrus blight and declinio (Beretta et al., 1988). Callose deposition is mediated by the callose synthase situated in the plasma membrane and mature xylem vessels do not exhibit a plasma membrane. It is thus likely that callose is synthesized by vessel-associated cells. In trees, it has been shown that vessel-associated cells synthetize carbohydrates that are deposited in the xylem vessels and contribute to cold hardening (Améglio et al., 2004) or bud break (Bonhomme et al., 2010).

## Conclusion

Our results demonstrate a clear temporal association between the accumulation of gel in the xylem lumens, the reduction in xylem hydraulic conductance, the grain dehydration and the cessation of grain dry mass accumulation. It is therefore tempting to postulate that beyond this succession of events, there is a causal relationship with gel deposition in the xylem lumens acting as a triggering mechanism that will eventually cause the end of the grain-filling phase. This hypothesis needs to be tested in future work. Similar decreases in xylem hydraulic conductance at the latter stages of fruit development have been reported in fleshy fruits like grape (Tyerman et al., 2004; Choat et al., 2009; Knipfer et al., 2015), tomato (*Solanum lycopersicum*; Malone and Andrews, 2001; Van Ieperen et al., 2003), or apple (*Malus domestica*; Lang and Ryan, 1994). As in the present study, the decrease in xylem hydraulic conductance in the whole berry and receptacle in the latter stage of ripening was associated with the deposition of gels or solutes in xylem conduits (Choat et al., 2009). This suggests that xylem hydraulics plays a key role in the mechanism of fruit and grain development and provide the basis for further work into the physiological molecular mechanisms responsible for the blockage of ear rachis xylem vessels of maturing wheat grains. Future work should also determine if a similar blockage of xylem vessels occurs during grain maturation in other Poaceae species.

## Author Contributions

HN, carried out the experiments, analyzed the data and edited the article; PM and HC, designed the research and drafted the manuscript; NB, performed the anatomical study, analyzed the results and edited the article.

## Conflict of Interest Statement

The authors declare that the research was conducted in the absence of any commercial or financial relationships that could be construed as a potential conflict of interest.
